# Accurate Localization Method Combining Optimized Hybrid Neural Networks for Geomagnetic Localization with Multi-Feature Dead Reckoning

**DOI:** 10.3390/s25051304

**Published:** 2025-02-20

**Authors:** Suqing Yan, Baihui Luo, Xiyan Sun, Jianming Xiao, Yuanfa Ji, Kamarul Hawari bin Ghazali

**Affiliations:** 1 Guangxi Key Laboratory of Precision Navigation Technology and Application, Guilin University of Electronic Technology, Guilin 541004, China; yansuqing@guet.edu.cn; 2 School of Information and Communication, Guilin University of Electronic Technology, Guilin 541004, China; 22022303095@mails.guet.edu.cn; 3 National & Local Joint Engineering Research Center of Satellite Navigation Localization and Location Service, Guilin 541004, China; sunxiyan@163.com (X.S.); jiyuanfa@163.com (Y.J.); 4 School of Science and Technology, Guilin University, Guilin 541006, China; 5 Center for Advanced Industrial Technology, University Malaysia Pahang Al Sultan Abdullah, Pekan 26600, Malaysia; kamarul@umpsa.edu.my

**Keywords:** indoor localization, particle swarm optimization, hierarchical BiLSTM, heading estimation, dead reckoning

## Abstract

Location-based services provide significant economic and social benefits. The ubiquity, low cost, and accessibility of geomagnetism are highly advantageous for localization. However, the existing geomagnetic localization methods suffer from location ambiguity. To address these issues, we propose a fusion localization algorithm based on particle swarm optimization. First, we construct a five-dimensional hybrid LSTM (5DHLSTM) neural network model, and the 5DHLSTM network structure parameters are optimized via particle swarm optimization (PSO) to achieve geomagnetic localization. The eight-dimensional BiLSTM (8DBiLSTM) algorithm is subsequently proposed for heading estimation in dead reckoning, which effectively improves the heading accuracy. Finally, fusion localization is achieved by combining geomagnetic localization with an improved pedestrian dead reckoning (IPDR) based on an extended Kalman filter (EKF). To validate the localization performance of the proposed PSO-5DHLSTM-IPDR method, several extended experiments using Xiaomi 10 and Hi Nova 9 are conducted in two different scenarios. The experimental results demonstrate that the proposed method improves localization accuracy and has good robustness and flexibility.

## 1. Introduction

Location-based services (LBSs) have received increased attention because of their high social requirements. Accurate location in intelligent navigation positioning systems can provide effective support for optimizing travel routes, improving traffic efficiency, and so on. Merchants can supply precise marketing and push personalized promotional activities according to location services. LBSs are also the core part of the development of smart cities and have broad development prospects [1]. However, satellite signals cannot penetrate indoor environments because of building obstructions; consequently, the global navigation satellite system (GNSS) cannot be engaged [2]. Therefore, indoor positioning technology research has become an extremely important research hotspot.

Researchers and scholars have conducted a series of studies on indoor positioning techniques and have made great achievements. However, indoor positioning technology still faces challenges. Among the available technologies. Among the existing technologies, such as Wi-Fi positioning [3], Bluetooth positioning [4], ultra-wideband (UWB) positioning [5], and pseudo-satellite positioning [6]. These techniques, while highlighting the advantages of relatively good positioning, also have drawbacks that are hard to ignore. In Wi-Fi positioning, its signal is easily interrupted by the complex indoor environment, with obvious multipath effects, and when the environment changes dynamically, such as intensive indoor personnel movement or layout changes, the positioning accuracy will drop drastically. In Bluetooth positioning, signal coverage is limited, generally only within a few tens of meters, and a large scene deployment of Bluetooth beacons will be costly. In UWB positioning, accuracy can reach a centimeter level, but the serious signal delay and distortion in non-line-of-sight propagation, the high cost of equipment, and the poor compatibility of equipment from different manufacturers limit large-scale applications. Pseudo-satellite positioning faces the problem of difficult signal synchronization and the difficulty and high cost of deployment and commissioning. There are still no unified technical specifications or mature applications for indoor positioning technologies. Geomagnetic signals are very pervasive and stable, and they are able to penetrate a wide range of building materials without the need for additional infrastructure, so low-cost positioning can be realized based on geomagnetic positioning technology.

Geomagnetism is a natural phenomenon that occurs within the Earth’s interior. In addition, many iron-containing materials cause geomagnetic anomalies in indoor buildings, and anomalous geomagnetism with more features is beneficial for positioning. Moreover, geomagnetic positioning can achieve low cost and consumer-level accuracy. It has broad application prospects. In geomagnetic positioning methods, dynamic time warping (DTW) [7] is the classic algorithm. Geomagnetic positioning based on DTW seeks geomagnetic similarity by finding the minimum cumulative distance between the geomagnetic sequence to be located and the geomagnetic fingerprint database, and the target location can be determined. Subbu et al. [8] adopted the DTW algorithm to achieve target localization of different motion speeds, which can effectively mitigate the impact of different states and achieve a positioning accuracy of 2–6 m. Gong et al. [9] proposed a geomagnetic localization algorithm using PDR-assisted DTW. The presented method can achieve reliable and continuous positioning and meet consumer-level positioning requirements. Qiu et al. [10] combined DTW with the Spearman correlation coefficient algorithm to achieve target positioning and reached a positioning accuracy of approximately 1 m. Another widely used geomagnetic-based localization method is the particle filter. It can be used in nonlinear and non-Gaussian distribution states and has strong generalizability. Xie et al. [11] presented a geomagnetic positioning method with an improved particle filter and achieved an average positioning accuracy of 1~2.8 m. Shu et al. [12] developed a bidirectional particle filter to fuse indoor geomagnetic and Wi-Fi signals. It can reach a positioning error of 3.5 m in indoor environments and effectively improve positioning accuracy.

Inertial sensor-based dead recognition can achieve accurate positioning in a short time. However, cumulative errors may occur over time. Pedestrian dead reckoning (PDR) is a relative positioning algorithm that is often combined with other algorithms in positioning. The PDR method includes step detection, step length estimation, heading angle estimation, and dead reckoning. Khedr et al. [13] proposed a step correction method that can increase PDR reliability in the long term. Gusenbauer et al. [14] proposed a localization method that combines PDR based on a linear step model with map matching, and its positioning performance can reach 3.6 m. Wang et al. [15] proposed a positioning method that adopts a floor plan to assist in identifying headings. It can achieve a positioning accuracy of 2.5 m.

Overall, DTW is a commonly used geomagnetic localization algorithm. It can achieve signal analysis at different speeds, ensure the acquisition of a global optimal solution, and improve the accuracy of geomagnetic similarity calculations. In addition, DTW handles nonlinear, related time sequences without specific data distributions or model assumptions. However, DTW has high time and space computational costs and is not suitable for real-time applications or large-scale datasets. Insufficient boundary conditions may lead to overfitting. In symmetrical and approximate indoor environments, due to the small difference in the geomagnetic field, significant positioning errors resulting from the adoption of DTW may occur due to geomagnetic blurring. Indoor geomagnetic fields are usually characterized by nonlinear and non-Gaussian distributions, and particle filtering can effectively address this complex state space, making it a commonly used localization method. It has wide applicability, multimodal processing ability, and strong flexibility. However, high computational complexity, particle degradation, and strong initial state dependence occur in positioning processing. Even when resampling techniques are used, some particles with small weights are generated, which can affect the localization performance. Using only geomagnetic positioning may result in positional ambiguity, which can lead to lower positioning accuracy. Therefore, a single indoor positioning technology cannot achieve high precision.

To address these issues, a positioning method, PSO-5DHLSTM-IPDR, is proposed. In this method, the particle swarm optimization algorithm is used to optimize the parameters in the 5DHLSTM model, and the stability and accuracy of the 5DHLSTM model can be improved. For PDR positioning, an 8DBiLSTM is proposed for heading estimation, in which the model uses eight-dimensional feature data for training and prediction to improve the heading accuracy. Because PDR can obtain reliable location information in a short time, while geomagnetic positioning can maintain relatively stable accuracy over a long period of time, combining geomagnetic positioning and PDR creates a complementary advantage, thus providing more accurate and reliable positioning for smartphones. The main contributions of this paper are as follows:

**PSO-5DHLSTM neural network model:** We propose a PSO-5DHLSTM neural network model for geomagnetic localization. First, the structure of the 5DHLSTM network is constructed, and the particle swarm is iteratively optimized according to the value of the fitness function. Afterward, the optimal parameters are used as the parameters of the 5DHLSTM network. Finally, the PSO-5DHLSTM neural network model can achieve target location prediction on the basis of geomagnetic sequences. The experimental results show that the geomagnetic positioning accuracy is improved and that the influence of different devices on the positioning accuracy is reduced.

**Heading angle estimation based on 8DBiLSTM**: To extract more accurate features, we constructed an eight-dimensional dataset. A hierarchical BiLSTM model is designed in this paper. Experiments show that the proposed 8DBiLSTM neural network can achieve more accurate heading estimation.

**A PSO-5DHLSTM-IPDR fusion localization method:** A PSO-5DHLSTM-IPDR fused localization system is proposed. An extended Kalman filter (EKF) is adopted to achieve fusion localization. The magnetic estimation is used as the measurement vector, and the dead reckoning estimation is used as the state vector. Two scenarios were subjected to experimental investigation, the results of which indicate that the proposed localization system is more accurate than the comparison algorithms are.

The rest of the paper is constructed as follows: Related works are introduced in Section 2. In Section 3, the materials and methodology are presented. Section 4 describes the experimental results. Finally, the paper is concluded in Section 5.

## 2. Related Works

A series of geomagnetic positioning studies have been carried out. Han et al. [16] constructed a fingerprint library with magnetic sequences and employed a recurrent neural network (RNN) to achieve indoor localization via geomagnetism. Bhattarai et al. [17] proposed a deep recursive neural network (DRNN) for geomagnetic localization, which can extract features of variable-length input sequences. Lee et al. [18] proposed accurate magnetic indoor localization (AMID) via deep learning, which effectively identifies magnetic sequence features. Ashraf et al. [19] proposed the use of a convolutional neural network (CNN) for indoor localization. Zhang et al. [20] presented indoor geomagnetic localization based on a long short-term memory (LSTM) network model. Yang et al. [21] presented a localization method that integrates 5G wireless signals and geomagnetic sequences. An error backpropagation neural network (BPNN) model was adopted to achieve more reliable localization performances. Shu et al. [22] presented a multiscale recurrent neural network (DM-RNN) to achieve indoor localization. Ding et al. [23] designed a CNN-LSTM neural network model for indoor localization. This model can automatically learn and match magnetic sequences with fingerprint databases. He et al. [24] proposed the ST-Loc geomagnetic localization system. Wang et al. [25] proposed a novel deep learning and data-feature augmentation-based magnetic localization framework (DarLoc). Ni et al. [26] proposed a geomagnetic indoor localization-based dilated convolution neural network (DCGIL). The accuracy of the DCGIL method was improved by using an expanded convolutional neural network for fingerprint segmentation classification.

Some researchers have used machine learning or deep learning methods to gain heading estimation in dead recognition. Liu et al. [27] proposed an LSTM neural network model to estimate the heading angle. Wang et al. [28] proposed a new type of heading estimation method based on spatial Transformer networks (STNs) and LSTM. Zhang et al. [29] presented a novel online extreme learning machine (OS-ELM) method. Huang et al. [30] proposed an LSTM-based heading error compensation model to effectively mitigate the heading error caused by gyroscope drift. Im et al. [31] utilized a multiple-layer neural network model to regress pedestrian headings.

Single-source positioning systems cannot balance various performance requirements, and multisource fusion localization has become a popular research topic. Ma et al. [32] proposed a basic positioning system that adopted a hidden Markov model (HMM) that combines geomagnetic positioning with PDR. Kuang et al. [33] proposed a localization method that fuses inertial sensors and geomagnetic sequences. Hang et al. [34] adopted a conditional random field (CRF) model to fuse the PDR and geomagnetic information and achieve trajectory prediction via the Viterbi algorithm. The method improves the convergence time in the localization process and the positioning accuracy. Deng et al. [35] presented a fusion localization algorithm based on the EKF, which combines Wi-Fi and PDR localization. The experiments show that the algorithm improves the localization performance without increasing algorithm complexity. Karamat et al. [36] proposed a positioning method that combines computer vision and unmanned vehicle-guided inertial sensors. Sun et al. [37] proposed a fusion localization algorithm that combines Wi-Fi and PDR. Schatzberg et al. [38] proposed a fusion positioning algorithm that combines Wi-Fi with dead reckoning, which effectively suppresses the cumulative errors caused by inertial navigation and improves positioning performance. Wang et al. [39] presented an improved EKF algorithm using sigma points, which can significantly increase the localization accuracy.

Motivation by deep learning, a PSO-5DHLSTM-IPDR fusion localization method that utilizes deep learning is proposed. A PSO-5DHLSTM network model is constructed for geomagnetic localization. The particle swarm algorithm is used to optimize the 5DHLSTM model and select suitable parameters to construct the 5DHLSTM model to improve the performance of the 5DHLSTM model and improve the geomagnetic positioning accuracy. For PDR localization, 8DBiLSTM is proposed for heading estimation. This improves the accuracy of heading estimation. Finally, geomagnetic and PDR fusion uses the EKF for localization to improve the positioning accuracy.

## 3. Materials and Methodology

### 3.1. Overview

**Figure 1:**
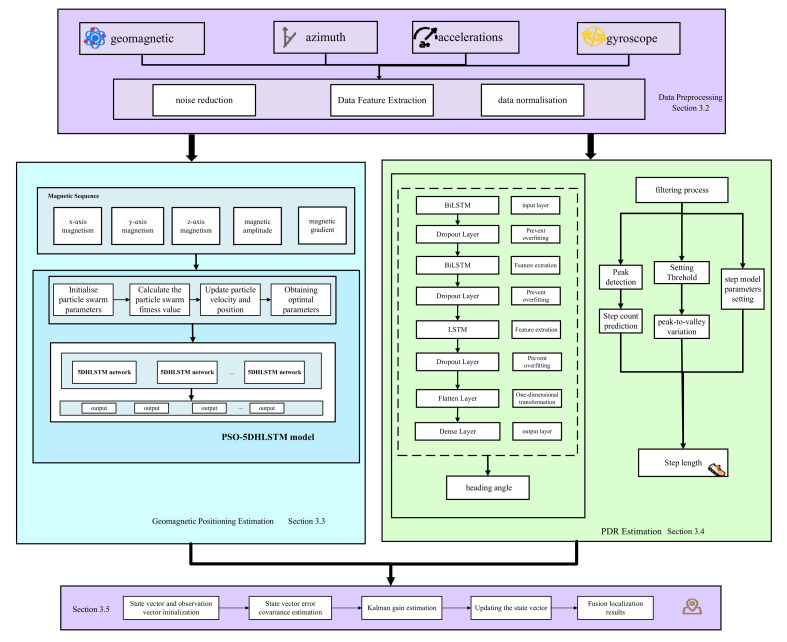
Overall structure of the fusion localization algorithm.

The general structure of the proposed PSO-5DHLSTM-IPDR localization method is shown in Figure 1. In this proposed framework, there are four models: data preprocessing, the PSO-5DHLSTM network, dead reckoning estimation, and fusion localization.

**Data preprocessing model:** Due to the interference of noise during magnetic sequences and IMU data collection processing, data preprocessing reduces the interference of noise with the data.

**PSO-5DHLSTM model:** The particle swarm parameters are first initialized, and the particles are randomly generated. Moreover, we construct the 5DHLSTM network model. Let the particle swarm initialize values as the neural network’s initial parameters; the fitness function value of each particle can be achieved through iterative optimization. Finally, the determined optimal parameters are fed into the trained 5DHLSTM model, and the location prediction based on magnetic sequences can be completed.

**Dead reckoning estimation:** Dead reckoning based on an inertial measurement unit includes step length estimation, heading angle estimation, and location updates. For dead reckoning estimation, an 8DBiLSTM network model is proposed to predict the heading angle.

**Fusion localization model:** In this model, the dead reckoning estimation is used as the state variable, and the PSO-5DHLSTM estimation is used as the measurement variable. Finally, fusion localization is achieved through an extended Kalman filter.

The flow of the proposed PSO-5DHLSTM-IPDR fusion positioning method is depicted in Algorithm 1.
**Algorithm 1:** A PSO-5DHLSTM-IPDR fusion localization method**Input:** Geomagnetic and inertial measurement unit data.**Output:** Target location prediction. 1: Data collection from cell phones. 2: Geomagnetic data preprocessing in Section 3.2. 3: //Geomagnetic localization estimation process. 4: Setting the Particle Swarm Parameters in Section 3.3. 5: Constructing the 5DHLSTM model in Section 3.3. 6: Calculating per-particle fitness values in Section 3.3. 7: Optimization of the model parameters in Section 3.3. 8: Geomagnetic localization using PSO-5DHLSTM models in Section 3.3. 9: // Dead reckoning estimation process. 10: Step length estimation in Section 3.4. 11:// Heading angle estimation process. 12: Heading angle data preprocessing in Section 3.4. 13: Constructing an 8DBiLSTM heading estimation model in Section 3.4. 14: Heading angle estimation using the 8DBiLSTM model in Section 3.4. 15: Dead reckoning estimation. 16: EKF algorithm parameter initialization. 17: **for** each step **do**. 18:  Initializing the equation of state and observation equations by Equation (25). 19:  The forecast covariance is obtained by Equation (33) 20:  Obtaining the Kalman gain by Equation (34). 21:  Updating the state equation by Equation (35). 22:  Updating the covariance equation through Equation (36). 23:  Saving the value of each status update. 24: **end for** 25: Getting the predicted location.

### 3.2. Data Preprocessing

Geomagnetic fields are widespread in nature. Moreover, in indoor environments, geomagnetic anomalies occur due to reinforced concrete, electrical wiring, iron furniture, electronic equipment, etc., in buildings. Anomalies generated by structural disturbances in indoor buildings can constitute special geomagnetic fingerprint features that make the location recognizable.

**Figure 2:**
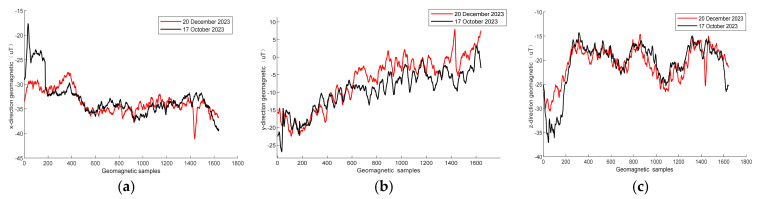
Geomagnetic signals at the same location on 20 December 2023 and 17 October 2023: (**a**) geomagnetic x-direction vector; (**b**) geomagnetic y-direction vector; (**c**) geomagnetic z-direction vector.

Geomagnetic positioning reliability depends on a stable indoor magnetic field. To verify the stability of the indoor magnetic field, experiments were carried out in a 32 m corridor that has several fire hydrants and other ferrous materials. Figure 2 shows the triaxial geomagnetic components at different times. The results demonstrate that the amplitudes of the geomagnetic sequences at different times are basically consistent. Therefore, the geomagnetic field has good stability.

**Figure 3:**
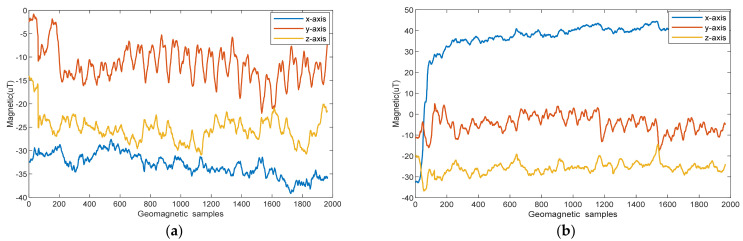
Geomagnetism captured using a mobile phone with different postures: (**a**) Pose 1; (**b**) Pose 2.

Owing to inconsistencies in the carrier and navigation coordinate systems, different acquisition attitudes can have an impact on the acquired magnetic field. Figure 3 shows the triaxial geomagnetic components acquired with different mobile phone postures in the 32 m corridor. The cell phone in Figure 3a is placed horizontally facing north (Pose 1). In Figure 3b, the cell phone is placed vertically with the screen facing north (Pose 2).

Figure 3 shows that the variation in the mobile phone orientation affects the geomagnetic distribution. Large deviations exist in the geomagnetic sequences acquired at different attitudes. Therefore, the geomagnetic modulus is calculated as

(1) $$M_{a}=\sqrt{M_{x}^{2}+M_{y}^{2}+M_{z}^{2}}$$

where $M_{x}$, $M_{y}$, and $M_{z}$ denote the triaxial magnetic values, and $M_{a}$ is the geomagnetic magnitude.

**Figure 4:**
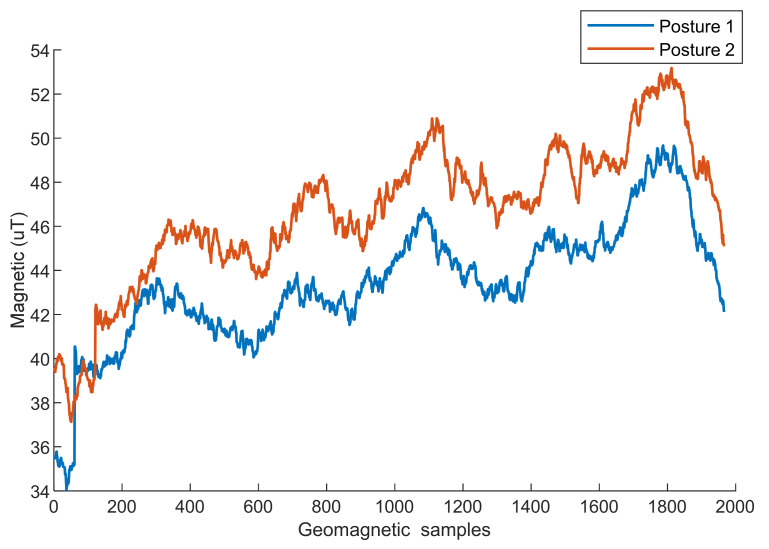
Geomagnetic magnitudes of different postures with the same phone.

Figure 4 shows the geomagnetic magnitude calculated with Equation (1) for Poses 1 and 2. The geomagnetic sequences of the two different attitudes have similar magnitudes. Therefore, the geomagnetic magnitude can be used as a geomagnetic eigen to eliminate the effects caused by different postures.

Geomagnetic data are disturbed by noise during the acquisition process. The empirical mode decomposition (EMD) [40,41] method is adopted to eliminate noise.

Specifically, the upper and lower envelopes of the original sequence are calculated via cubic spline interpolation after all local extrema from each magnetic sequence are obtained. The first new sequence $h_{1}$ is obtained by subtracting the average $e_{1}$ of the upper and lower envelopes from the original sequence mg(t). The formula is depicted as follows:

(2) $$mg(t)-e_{1}=h_{1}$$

The standard deviation (SD) is used to determine if the sequence $h_{1n}$ is an intrinsic mode function (IMF). If not, it will continue as a new input sequence until $h_{1n}$ is an intrinsic mode function.

(3) $$SD=\sum_{t=0}^{T}\left[\frac{|(h_{1(n-1)}(t)-h_{1n}(t){|}^{2}}{h_{1(n-1)}^{2}(t)}\right]$$

(4) $$h_{1(n-1)}-e_{1n}=h_{1n}$$

where $h_{1n}$ is an IMF, such that $im_{1}=h_{1n}$, $im_{1}$ is the first IMF. We can separate $c_{1}$ from the sequence mg(t), as shown in Equation (5). Because residue $res_{1}$ still contains information on the longer period component, it is viewed as new data and selected in the same way as above. The result is shown in Equation (6).

(5) $$mg(t)-im_{1}=res_{1}$$

(6) $$res_{1}-im_{2}=res_{2},\ldots res_{n-1}-im_{n}=res_{n}$$

The decomposition is completed until the last remaining residue *r_n_* is a monotonic function, a trend, or a constant function with an extreme value number less than 2. Finally, a residual component is obtained with a set of IMF data. From Equations (5) and (6), the following equations can be derived.

(7) $$mg(t)=\sum_{i=1}^{N}im_{i}(t)+res(t)$$

where N is the number of IMFs, ${\mathrm{im}}_{i}(t)$ is the *i-th* IMF, and res(t) is the nonzero-mean residual sequence.

For a uniform scale and range of magnitude sequences, Z score normalization is used to process the magnetic data as follows:

(8) $$Nor=\frac{M_{a}-\mu_{mag}}{\sigma_{mag}}$$

where $\mu_{mag}$ is the average of the geomagnetic sequence, $\sigma_{mag}$ is the standard deviation of the geomagnetic sequence, and Nor represents normalized geomagnetism.

To improve the recognizability of geomagnetism, we utilize multi-dimensional features for geomagnetic positioning. Three-axis geomagnetic data can be collected by a smartphone, and in addition, richer magnetic feature information can be synthesized using three-axis magnetic fields such as magnetic field strength and horizontal components. They are relatively stable and directionally independent of the smartphone, thus effectively eliminating device heterogeneity. The gradient and horizontal magnetic strengths are as follows:

(9) $$M_{d}=\left(\frac{\partial M_{x}}{\partial x}\right)e_{x}+\left(\frac{\partial M_{y}}{\partial y}\right)e_{y}+\left(\frac{\partial M_{z}}{\partial z}\right)e_{z}$$

(10) $$M_{h}=\sqrt{M_{x}^{2}+M_{y}^{2}}$$

where $\frac{\partial M_{x}}{\partial x}$, $\frac{\partial M_{y}}{\partial y}$, $\frac{\partial M_{z}}{\partial z}$ are the gradients of the geomagnetic field along the x-, y-, and z-axes, and $e_{x}$, $e_{y}$, $e_{z}$ are the unit vectors on each of the three axes.

We construct six-dimensional geomagnetic data for geomagnetic localization.

(11) $$Fea=[M_{x},M_{y},M_{z},M_{a},M_{d},M_{h}]$$

where $M_{d}$ is gradient, and Fea are six-dimensional geomagnetic data.

Finally, a uniformly sized sliding window is used to segment the geomagnetic sequence into several consecutive sub-sequences. The proposed localization method predicts the position of the target by matching the corresponding position of each frame in the sub-sequence.

### 3.3. PSO-5DHLSTM Model

**Figure 5:**
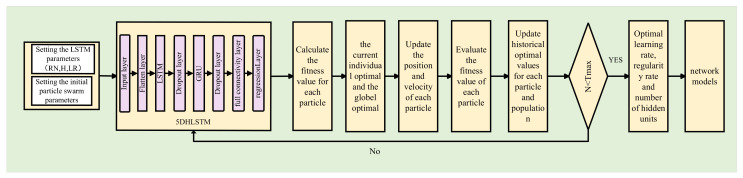
PSO-5DHLSTM model.

In existing deep learning research, network structure parameters such as the initial learning rate, number of hidden layer units, and regularization factor play key roles in localization performance. Empirical values or multiple trial values are adopted to initialize the neural network parameters. However, it is difficult to achieve optimal performance for different scenarios. To address this issue, we propose a novel particle optimization hybrid neural network model, as shown in Figure 5. This algorithm first calculates the fitness function through multiple iterations of particle swarm optimization training to achieve the optimal neural network parameters.

In the proposed particle optimization hybrid neural network model, each particle has two metrics: velocity and position. The velocity determines the direction and displacement of the particle’s next motion. The position describes the current position of the particle. The *i*-th particle in 3-dimensional space can be expressed as follows:

(12) $$\left\{\begin{array}{c} Pos_{i}=(pos_{i1},pos_{i2},pos_{i3})\\ Vel_{i}=(vel_{i1},vel_{i2},vel_{i3}) \end{array}\right.$$

where $Pos_{i}$ and $Vel_{i}$ denote the position and velocity of the *i-th* particle, respectively.

PSO calculates the particle fitness value and compares it with the particle’s historical optimal fitness value $gbest_{i}$ and the global optimal fitness value $zbest_{i}$ expressed in Equation (13).

(13) $$\begin{array}{l} \left\{\begin{array}{l} gbest_{i}=(gbest_{i1},gbest_{i2},gbest_{i3})\\ zbest_{i}=(zbest_{i1},zbest_{i2},zbest_{i3}) \end{array}\right. \end{array}$$

During the iterative process, the velocity and position of the particles are continuously updated to achieve the global optimum. The steps are as follows:

A. Particle population initialization

The three dimensions of this particle $Pos_{i}$ can be set to these three parameters (RN,H,LR), where RN is the regular rate of the 5DHLSTM neural network, *H* is the number of neurons in the hidden layer, and LR is the neural network learning rate.

The parameters to be initialized in this algorithm include the maximum number of iterations $T_{\max}$ = 10, the inertia weight w = 0.5, and the learning factors $\delta_{1}$ = 2 and $\delta_{2}$ = 2.

B. Hybrid neural network structure construction.

The long short-term memory (LSTM) neural network is an extension of the RNN and can store and transfer information. The LSTM neural network module is composed of an input gate, an output gate, and a forget gate. Input gates pass information from the input layer to the implicit layer. Forget gates control how previously stored information is forgotten so that important information can be selectively retained and unimportant information discarded. The output gate is responsible for passing the information. Geomagnetism is a kind of time series data; LSTM can handle this kind of geomagnetic sequence data well and can effectively capture the long-term dependency and time series features in geomagnetic data to more accurately analyze and utilize the geomagnetic data for localization. The expression of LSTM is as follows:

(14) $$\left\{\begin{array}{l} e_{mag}^{t}=\sigma (W_{e_{mag}}\cdot [h_{mag}^{t-1},s_{mag}^{t}]+b_{e_{mag}})\\ d_{mag}^{t}=\sigma (W_{d_{mag}}\cdot [h_{mag}^{t-1},s_{mag}^{t}]+b_{d_{mag}})\\ o_{mag}^{t}=\sigma (W_{o_{mag}}\cdot [h_{mag}^{t-1},s_{mag}^{t}]+b_{o_{mag}})\\ g_{mag}^{t}=\tanh(W_{z_{mag}}\cdot [h_{mag}^{t-1},s_{mag}^{t}]+b_{z_{mag}})\\ z_{mag}^{t}=f_{mag}^{t}\cdot z_{mag}^{t-1}+e_{mag}^{t}\cdot g_{mag}^{t} \end{array}\right.$$

where $e_{mag}^{t}$, $d_{mag}^{t}$, and $o_{mag}^{t}$ are the input, forget, and output gates, respectively. $z_{mag}^{t}$ is a memory cell from a previous moment, and $g_{mag}^{t}$ is a candidate for memory updating. $h_{mag}^{t-1}$ is the hidden state. $s_{mag}^{t}$ is the input feature, and σ and tanh are sigmoid and hyperbolic tangent functions, respectively. $W_{e_{mag}}$, $W_{d_{mag}}$, $W_{o_{mag}}$, and $W_{z_{mag}}$ are the weights. $b_{e_{mag}}$, $b_{d_{mag}}$, $b_{o_{mag}}$, and $b_{z_{mag}}$ are the biases.

The GRU is an improved LSTM network and has a simpler neuron structure, fast training speed, and high efficiency. It is more suitable for dynamic process modeling. The GRU model includes only two gates, the update gate and the reset gate, as shown in Equation (15):

(15) $$\left\{\begin{array}{l} u_{mag}^{t}=\sigma (W_{u_{mag}}\cdot [q_{mag}^{t-1},s_{mag}^{t}]+b_{u_{mag}})\\ r_{mag}^{t}=\sigma (W_{r_{mag}}\cdot [q_{mag}^{t-1},s_{mag}^{t}]+b_{r_{mag}})\\ {\tilde{q}}_{mag}^{t}=\tanh(W_{q_{mag}}\cdot [u^{t}*q_{mag}^{t-1},s_{mag}^{t}]+b_{q_{mag}})\\ q_{mag}^{t}=(1-r_{mag}^{t})*q_{mag}^{t-1}+r_{mag}^{t}*{\tilde{q}}_{mag}^{t} \end{array}\right.$$

where $u_{mag}^{t}$ and $r_{mag}^{t}$ are the update gate and reset gate, respectively. $q_{mag}^{t-1}$ is the state from the previous time step. ${\tilde{q}}_{mag}^{t}$ is a candidate state for the hidden layer. $W_{u_{mag}}$ and $W_{r_{mag}}$ are the weights of two different gates, and $W_{q_{mag}}$ is the weight of the cell states. $b_{u_{mag}}$ and $b_{r_{mag}}$ are the biases of two different gates, and $b_{q_{mag}}$ is the bias of the cell state.

We construct the 5DHLSTM model with eight layers. The first layer is the input layer used for acquiring geomagnetic data. The second layer is the flatten layer, which is used for transforming multidimensional data into one-dimensional data. The third and fifth layers used for geomagnetic feature extraction are the LSTM layer and the GRU layer, respectively. The fourth and sixth layers are exit layers with an exit rate of 0.2 to avoid overfitting. The fully connected layer and regression layer ensure that the geomagnetic features are not lost to achieve precise positioning.

C. Particle fitness function value calculation

The initial particles of each population are randomly generated for the 5DHLSTM neural network. To achieve the optimal parameters for the proposed hybrid neural network, the fitness function *f* of each particle needs to be calculated as follows:

(16) $$f=\frac{1}{N}\sum_{k=1}^{N}\sqrt{{\left(X_{pre}(k)-X_{true}(k)\right)}^{2}+{\left(Y_{pre}(k)-Y_{true}(k)\right)}^{2}}$$

where $\left(X_{pre},Y_{pre}\right)$ represent the predicted location of the target, and $\left(X_{\mathrm{true}},Y_{\mathrm{true}}\right)$ represent the true location of the target.

D. Neural network parameter optimization

According to Equations (17) and (18), the positions and velocities of the particles are updated as follows:

(17) $$Vel_{i}^{n+1}=wVel_{i}^{n}+\delta_{1}\alpha_{1}[gbest_{i}^{n}-Position_{i}^{n}]+\delta_{2}\alpha_{2}[zbest_{i}^{n}-Position_{i}^{n}]$$

(18) $$Position_{i}^{n+1}=Position_{i}^{n}+Vel_{i}\delta_{1}\alpha_{1}^{n+1}$$

where w, $\delta_{1}$, and $\delta_{2}$ have been described in the particle population initialization step; $\alpha_{1}$ and $\alpha_{2}$ are random numbers; $Vel_{i}^{n}$ is the particle velocity in the *n*-th iteration; $Position_{i}^{n}$ is the particle position in the *n*-th iteration; $gbest_{i}^{n}$ is the individual optimal position; and $zbest_{i}^{n}$ is the global optimal position.

After the update of the particle position and velocity is complete, the particle fitness function, particle global optimum, and per-particle optimum need to be recalculated. In this algorithm, we also set an adaptive function threshold to ensure positioning accuracy. When it is less than the threshold, the algorithm stops updating the particles and uses the current optimal parameters as the neural network parameters; otherwise, it returns to training the neural network.

E. Positioning prediction

The optimal parameters obtained via the previous step are input into the proposed neural network to forecast the target location.

To verify the localization performance of the designed PSO-5DHLSTM, experiments were conducted using different mobile phones in an underground car park (45.4 × 30.7 × 3.5 m^3^). Table 1 shows the root mean square errors between 5DHLSTM and PSO-5DHLSTM. The positioning error of PSO-5DHLSTM is smaller than that of 5DHLSTM. This is because the particle swarm is used to optimize the parameters of the 5DHLSTM to select the optimal combination of parameters to construct the 5DHLSTM model, which can increase the performance of the 5DHLSTM neural network and thus improve geomagnetic positioning accuracy.

**Figure 6:**
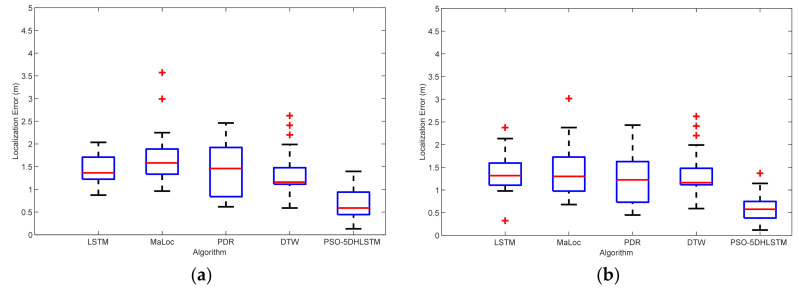
Localization errors of the LSTM, MaLoc, PDR, DTW, and PSO-5DHLSTM methods: (**a**) Xiaomi 10, (**b**) Hi Nova 9.

Figure 6 shows the localization errors of LSTM, Maloc, PDR, DTW, and PSO-5DHLSTM. The experimental results reveal that the PSO-5DHLSTM method has the smallest mean localization error, and the outliers are significantly reduced. This is because the proposed algorithm uses particle swarm optimization to obtain the optimal network parameters on the basis of the current data, which can be adapted to different positioning scenarios.

### 3.4. 8DBiLSTM Heading Angle Estimation Model

PDR position estimation consists of step detection, step length estimation, and heading estimation. Accurate heading estimation is crucial for pedestrian dead recognition. In the deep learning network, BiLSTM processes the inputs through two independent LSTM layers in chronological and reverse chronological order. It can capture the comprehensive features in the forward and reverse directions. During pedestrian’s continuous walking, the previous straight walking state and the subsequent turning movement will have an impact on the heading judgment at the current moment. BiLSTM can effectively integrate these features to provide a richer basis for heading estimation at each time step. It can learn the dependencies between different time points to better handle the continuity and correlation of heading changes. The hidden state ${\;h}_{\theta }$ and the cell state $c_{\theta }$ at each time step *t* are computed as follows:

(19) $$\left\{\begin{array}{l} i_{\theta }^{t}=\sigma (R_{i\theta }\cdot [h_{\theta }^{t-1},\tilde{H_{\theta }^{t}}])+a_{i\theta }\\ f_{\theta }^{t}=\sigma (R_{f\theta }\cdot [h_{\theta }^{t-1},\tilde{H_{\theta }^{t}}])+a_{f\theta }\\ o_{\theta }^{t}=\sigma (R_{o\theta }\cdot [h_{\theta }^{t-1},\tilde{H_{\theta }^{t}}])+a_{o\theta }\\ c_{\theta }^{t}=f_{\theta }^{t}*c_{\theta }^{t-1}+i_{\theta }^{t}*\tanh(R_{c\theta }\cdot [h_{\theta }^{t-1},\tilde{H_{\theta }^{t}}])+a_{c\theta })\\ h_{\theta }^{t}=o_{\theta }^{t}\tanh(c_{\theta }^{t}) \end{array}\right.$$

where $i_{\theta }^{t}$, $f_{\theta }^{t}$, and $o_{\theta }^{t}$ are the input gate, forget gate, and output gate, respectively. $c_{\theta }^{t}$ represents the candidate state. σ and tanh are sigmoid and hyperbolic tangent functions, respectively. $R_{i\theta }$, $R_{f\theta }$, $R_{o\theta }$, and $R_{c\theta }$ are the weights. $a_{i\theta }$, $a_{f\theta }$, $a_{o\theta }$, and $a_{c\theta }$ are the biases.

**Figure 7:**
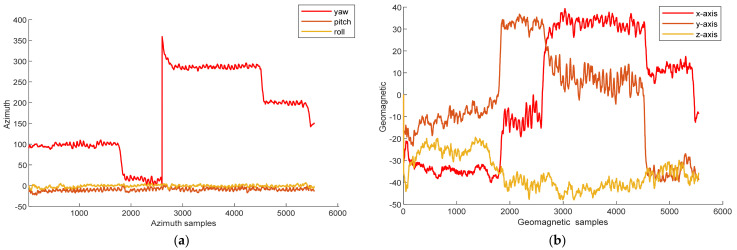
Data collected via mobile phone: (**a**) triaxial component of the azimuth; (**b**) triaxial component of the geomagnetic field.

For heading estimation, azimuth data and geomagnetic data were collected with a smartphone. Figure 7 shows three-axis azimuthal data and three-axis geomagnetic data in a gyratory corridor. The figure shows that when the heading changes, the magnitude of the geomagnetic and azimuthal angles significantly changes. Therefore, these data are related to the target heading.

In this paper, we construct an eight-dimensional dataset for heading estimation, as shown in Equation (20):

(20) $$H_{\theta }=[mag_{x},mag_{y},mag_{z},mag_{abs},ori_{x},ori_{y},ori_{z},ori_{abs}]$$

where $mag_{x}$, $mag_{y}$, $mag_{z}$, $ori_{x}$, $ori_{y}$, and $ori_{z}$ are the *x*, *y*, and *z* components of the geomagnetic and azimuthal angles, respectively. Then, $mag_{abs}$ and $ori_{abs}$ are the geomagnetic and azimuthal magnitudes, respectively, which can be calculated via Equations (21) and (22).

(21) $$mag_{abs}=\sqrt{mag_{x}^{2}+mag_{y}^{2}+mag_{z}^{2}}$$

(22) $$ori_{abs}=\sqrt{ori_{x}^{2}+ori_{y}^{2}+ori_{z}^{2}}$$

To reduce the impact of the heterogeneity of different mobile phone devices, the eight-dimensional dataset needs to be scaled to [0, 1], as defined below:

(23) $$\tilde{H_{\theta }}=\frac{H_{\theta }-\min(H_{\theta })}{\max(H_{\theta })-\min(H_{\theta })}$$

where $\min(H_{\theta })$ and $\max(H_{\theta })$ are the minimum and maximum values, respectively, of the dataset, and $\tilde{H_{\theta }}$ is the dataset after normalization.

After eight-dimensional feature dataset construction, we propose a hierarchical 8DBiLSTM model to mine the head features. The hierarchical 8DBiLSTM model has eight layers. The first layer is the input layer used for acquiring data. The second, fourth, and sixth layers are exit layers with an exit rate of 0.2 to avoid overfitting. The third and fifth layers are the BiLSTM layers for feature extraction. The seventh layer is the flatten layer used for transforming the multidimensional data into one-dimensional data. The dense layer is subsequently used to output the predicted values. The proposed heading estimation model can be described as follows:

(24) $$HD_{\theta }=NET_{H-BiLSTM}(D_{k}^{H_{\theta }})$$

where $NET_{H-BiLSTM}(D_{k}^{H_{\theta }})$ is the proposed network model, and $D_{k}^{H_{\theta }}$ is the result after data segmentation. $HD_{\theta }$ is heading prediction.

**Figure 8:**
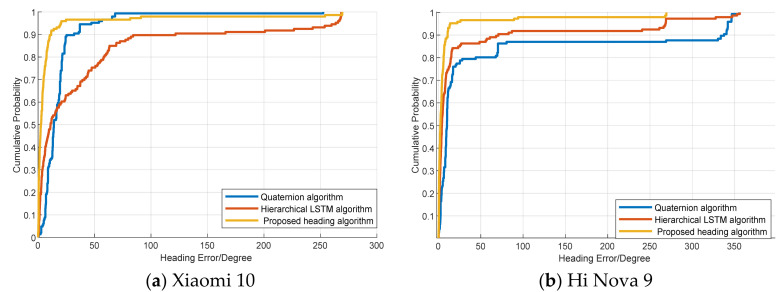
Cumulative distribution function of the heading error in Scene 1.

To validate the heading estimation performance, we compare the proposed method with hierarchical LSTM [27] and the quaternion method [42] using Xiaomi 10 (Beijing, China) and Hi Nova 9 (Guangdong, China)terminals in two different scenes (the two different scenes are described in Section 4.1). Figure 8 displays the cumulative distribution function (CDF) of the heading error with different terminals. The results indicate that the proposed method can achieve more accurate heading estimations. This is because the BiLSTM neural network model can mine more effective features from both the forward and backward directions, and this results in a more accurate heading performance.

**Figure 9:**
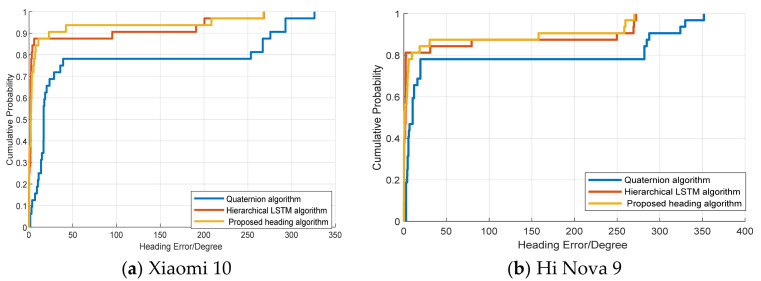
Cumulative distribution function of the heading error in Scene 2.

Figure 9 shows the CDF of the heading error among the proposed method, hierarchical LSTM, and quaternion methods in Scene 2. The experimental results demonstrate that our proposed heading estimation model achieves good accuracy in Scene 2. This is because the proposed algorithm can extract more accurate features and achieve better localization performance in more complex environments.

Table 2 and Table 3 present the mean errors and RMSEs among the different heading algorithms in the two scenarios. The proposed heading algorithm can achieve a mean error of approximately 10 °C and an RMSE of approximately 40 °C. The experimental results indicate that the proposed heading algorithm has stronger robustness in different scenes and different terminals.

### 3.5. EKF-Based Fusion Positioning

In this section, fusion positioning is achieved via an extended Kalman filter (EKF). The state vector $X_{state}$ and the measurement vector $Z_{measure}$ are depicted as follows:

(25) $$\left\{\begin{array}{l} X_{state}={\left[x_{pdr},y_{pdr},\varphi_{pdr},l_{pdr}\right]}^{T}\\ Z_{measure}={\left[x_{mag},y_{mag},\varphi_{mag},l_{mag}\right]}^{T} \end{array}\right.$$

where $\left(x_{pdr},y_{pdr}\right)$ indicates the location of the PDR estimation. $\varphi_{pdr}$ denotes the heading angle. $l_{mag}$ implies the step length. $\left(x_{mag},y_{mag}\right)$ denotes the location of geomagnetic positioning.

The state and observation equations are shown below:

(26) $$\left\{\begin{array}{l} X_{state}^{k}=F_{tm}^{k}(X_{state}^{k-1})+w_{state}^{k}\\ Z_{measure}^{k}=H_{tm}^{k}(X_{state}^{k-1})+v_{measure}^{k} \end{array}\right.$$

where $X_{state}^{k-1}$ indicates the previous moment of the state vector. $w_{state}^{k}$ and $v_{measure}^{k}$ are the state noise and measurement noise, respectively, which satisfy the normal distribution. $F_{tm}^{k}$ and $H_{tm}^{k}$ are nonlinear transition functions.

Taylor expansions of the nonlinear functions $F_{tm}^{k}(X_{state}^{k-1})$ and $H_{tm}^{k}(X_{state}^{k-1})$ around the prior estimation function $X_{state}^{k-1|k-1}$ are performed separately, retaining only the primary terms and ignoring the higher-order terms.

(27) $$X_{state}^{k}\approx F_{tm}^{k}[X_{state}^{k-1|k-1}]+\frac{\partial F_{tm}^{k}}{\partial X_{state}^{k-1|k-1}}[X_{state}^{k-1}-X_{state}^{k-1|k-1}]+w_{state}^{k}=\phi_{state}^{k-1}.X_{state}^{k-1}+w_{state}^{k}$$

(28) $$Z_{measure}^{k}\approx H_{tm}^{k}[X_{state}^{k-1|k-1}]+\frac{\partial H_{tm}^{k}}{\partial X_{state}^{k-1|k-1}}[X_{state}^{k-1}-X_{state}^{k-1|k-1}]+v_{state}^{k}=\psi_{measure}^{k-1}.X_{state}^{k-1}+v_{state}^{k}$$

The transformation matrix is obtained by taking the partial derivatives of the transformation function. The state transition matrix $\phi_{state}^{k}$ and measurement matrix $\psi_{measure}^{k}$ are expressed in Equation (29).

(29) $$\left\{\begin{array}{l} \phi_{state}^{k}=F_{tm}^{k\prime }=\frac{\partial F_{tm}^{k\prime }}{\partial X_{state}}\\ \psi_{measure}^{k}=H_{tm}^{k\prime }=\frac{\partial H_{tm}^{k\prime }}{\partial X_{state}} \end{array}\right.$$

The measurement matrix $\psi_{measure}^{k-1}$ is used to determine the state transfer matrix $\phi_{state}^{k-1}$.

(30) $$\phi_{state}^{k-1}=\left[\begin{array}{cccc} 1 & 0 & l_{pdr}\cdot \cos\varphi_{pdr}^{k-1} & -l_{pdr}\cdot \sin_{pdr}^{k-1}\\ 0 & 1 & l_{pdr}\cdot \sin\varphi_{pdr}^{k-1} & l_{pdr}\cdot \cos\varphi_{pdr}^{k-1}\\ 0 & 0 & 1 & 0\\ 0 & 0 & 0 & 1 \end{array}\right]$$

(31) $$\psi_{measure}^{k-1}=\left[\begin{array}{cccc} 1 & 0 & 0 & 0\\ 0 & 1 & 0 & 0\\ 0 & 0 & 1 & 0\\ 0 & 0 & 0 & 1 \end{array}\right]$$

The state is predicted, and the state at moment *k* is projected on the basis of the state at moment *k*-1, as shown in Equation (32):

(32) $$X_{state}^{k|k-1}=F_{tm}^{k}(X_{state}^{k-1|k-1})$$

The error covariance matrix $P_{error}^{k|k-1}$ and Kalman gain $K_{gain}^{k}$ are as follows:

(33) $$P_{error}^{k|k-1}=\phi_{state}\cdot P_{error}^{k-1|k-1}\cdot \phi_{state}^{T}+Q$$

(34) $$K_{gain}^{k}=\frac{P_{error}^{k|k-1}\cdot \psi_{measure}^{T}}{\psi_{measure}^{T}\cdot P_{error}^{k|k-1}\cdot \psi_{measure}^{T}+R}$$

where R is the noise covariance matrix of the observation vector.

The position is obtained on the basis of the Kalman gain, state values, and observations, as shown in Equation (35). Finally, $P_{error}^{k|k-1}$ is updated via the unit matrix *I* to obtain the covariance matrix $P_{error}^{k|k}$ to be used at the next time.

(35) $$X_{state}^{k|k}=X_{state}^{k|k-1}+K_{gain}^{k}\cdot (Z_{measure}^{k}-H_{tm}^{k}(X_{state}^{k|k-1}))$$

(36) $$P_{error}^{k|k}=(I-K_{gain}^{k}\cdot \psi_{measure})\cdot P_{error}^{k|k-1}$$

where $X_{state}^{k|k}$ is the state vector at moment *k,* and $X_{state}^{k|k-1}$ is one-step state vector prediction.

## 4. Experimental Results

In this section, Section 4.1 describes the experimental setting. The mean localization error of the proposed method is illustrated in Section 4.2. Section 4.3 presents the cumulative distribution function (CDF) of the proposed method. Section 4.4 presents the root mean square error and mean error of the localization algorithm.

### 4.1. Experimental Setting

**Figure 10:**
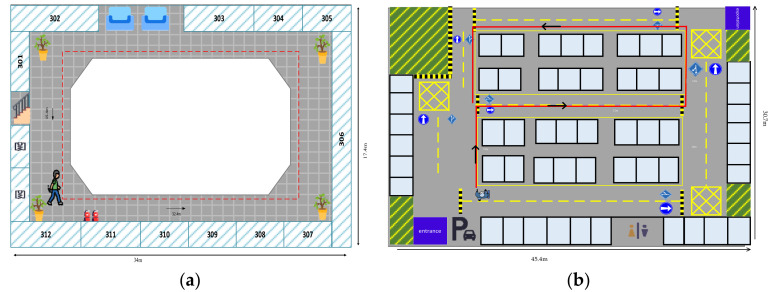
Floorplans of the experimental sites: (**a**) Scene 1; (**b**) Scene 2.

To validate the positioning performance of the presented method, extensive experiments were conducted in two different indoor scenarios, as shown in Figure 10. The first site is a rectangular corridor with dimensions of 34 × 17.2 × 5 m^3^, and the second scene is an underground parking lot with dimensions of 45.4 × 30.7 × 3.5 m^3^. The red lines in Figure 10 denote the planned paths. Path 1 is a 92 m rectangular corridor with firearms extinguishers, steel cabinets, balustrades, and some steel doors, as shown in Figure 10a. Path 2 is a 60 m continuous curved path with pipes and hydrants, as shown in Figure 10b.

The implementation of the proposed algorithm includes a server terminal and a client terminal. The server runs the Windows 10 64-bit operating system and has an Intel Core i7-7700H, and it is used to estimate the target location. Xiaomi 10 and Hi Nova 9 are adopted as the clients, which are used to collect data. Table 4 lists the specific parameters of these two smartphones.

We recruited two volunteers (#1 female 155 cm, #2 male 175 cm) from a local university to collect geomagnetic sequences and inertial measurement unit data from the acceleration and gyroscope sensors along the planned path. Moreover, the volunteers are required to capture data several times. The sampling frequency is 50 Hz.

### 4.2. Mean Localization Error

**Figure 11:**
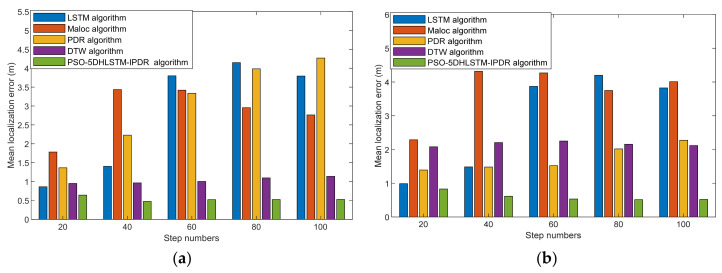
Average errors of LSTM, MaLoc, PDR, DTW, and the PSO-5DHLSTM-IPDR with different step numbers in Scene 1: (**a**) Xiaomi 10; (**b**) Hi Nova 9.

Figure 11 shows the average error of the different step numbers between LSTM [43], MaLoc [12], PDR [44], DTW [45], and the PSO-5DHLSTM-IPDR algorithm using Xiaomi 10 and Hi Nova 9 in Scene 1. The experimental results indicate that the average error of the PSO-5DHLSTM-IPDR algorithm is lower than those of the LSTM, MaLoc, PDR, and DTW algorithms when different terminals are used. The main reason is that the proposed PSO-5DHLSTM geomagnetic positioning algorithm can be obtained via a particle swarm to optimize the hybrid network model parameters. This allows the network model to better extract geomagnetic features. Geomagnetic positioning can be performed to achieve more accurate localization.

**Figure 12:**
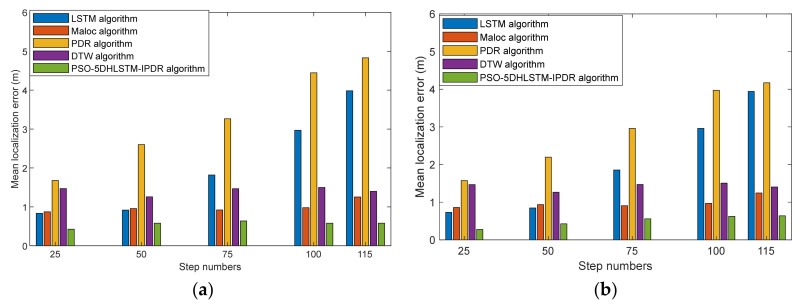
Average errors of LSTM, MaLoc, PDR, DTW, and the PSO-5DHLSTM-IPDR with different step numbers in Scene 2. (**a**) Xiaomi 10; (**b**) Hi Nova 9.

Figure 12 presents the average error of different step numbers in Scene 2. The PSO-5DHLSTM-IPDR algorithm also achieves comparable positioning accuracy in different scenes. This is because a particle swarm optimized 5DHLSTM model can extract more comprehensive geomagnetic features in different scenes and reduce the effect of device heterogeneity.

**Figure 13:**
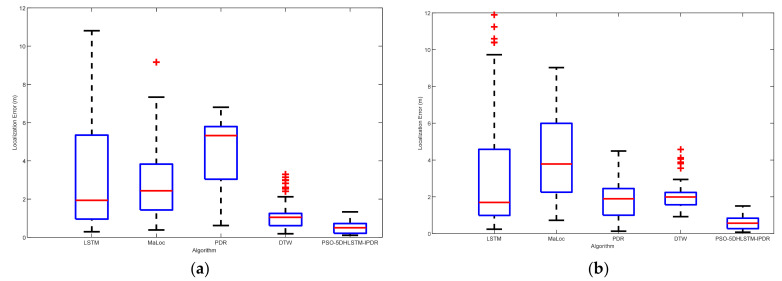
Localization errors of the LSTM, MaLoc, PDR, DTW, and PSO-5DHLSTM-IPDR methods in Scene 1 when different cell phones are used: (**a**) Xiaomi 10; (**b**) Hi Nova 9.

Figure 13 displays the localization errors of the LSTM, MaLoc, PDR, DTW, and the PSO-5DHLSTM-IPDR algorithms in Scene 1. The red crosses and red lines denote the outliers and average errors in the localization, respectively. Scene 1 has a ferromagnetic object in it, so it will be affected by it, thus causing a geomagnetic anomaly. The experimental results show that the mean and median of the positioning errors of the other algorithms are significantly shifted due to the influence of geomagnetic anomalies. The PSO-5DHLSTM-IPDR algorithm has the lowest average error and fewer anomalies than the other algorithms, as shown in Figure 13. This is because the presented algorithm can extract accurate features to overcome the geomagnetic anomalies and cumulative errors of PDR.

**Figure 14:**
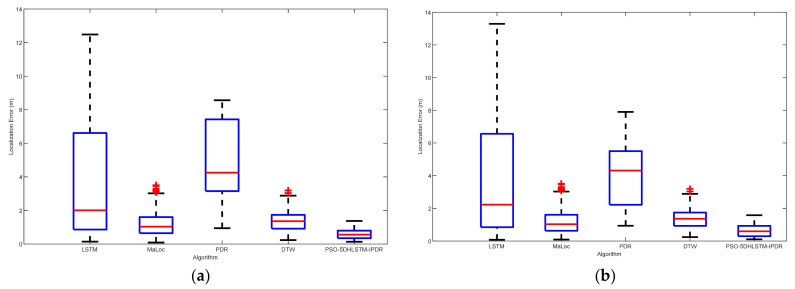
Localization errors of the LSTM, MaLoc, PDR, DTW, and PSO-5DHLSTM-IPDR methods in Scene 2: (**a**) Xiaomi 10; (**b**) Hi Nova 9.

Figure 14 illustrates the localization errors of the different algorithms in Scene 2. The results indicate that the PSO-5DHLSTM-IPDR method has the lowest localization error in Scene 2. The Experiments reveal that the presented algorithm effectively improves the localization performance, achieving comparable localization performance in more complex environments. This is because the proposed algorithm and the particle swarm optimization algorithm can be used to obtain the appropriate model parameters, which can lead to better performance of the network model to extract the geomagnetic features in different scenes.

### 4.3. Cumulative Distribution Function (CDF)

**Figure 15:**
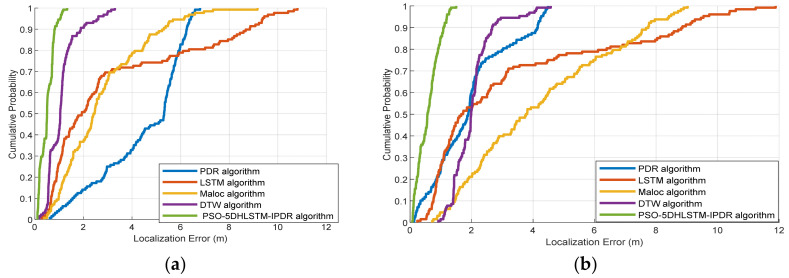
Cumulative distribution functions of the LSTM, MaLoc, PDR, DTW, and PSO-5DHLSTM-IPDR methods in Scene 1: (**a**) Xiaomi 10, (**b**) Hi Nova 9.

The CDFs of the localization errors obtained via the LSTM, MaLoc, PDR, DTW, and PSO-5DHLSTM-IPDR algorithms with Xiaomi 10 and Hi Nova 9 are shown in Figure 15. The experimental results demonstrate that the cumulative error of PSO-5DHLSTM-IPDR is less than 1 m when the cumulative probability error reaches 80%. This is because our proposed fusion localization system can effectively eliminate geomagnetism mismatching and alleviate the cumulative error of pedestrian dead reckoning.

**Figure 16:**
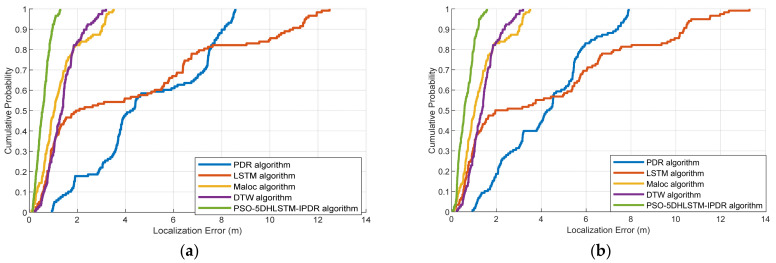
Cumulative distribution functions of the LSTM, MaLoc, PDR, DTW, and PSO-5DHLSTM-IPDR methods in Scene 2: (**a**) Xiaomi 10; (**b**) Hi Nova 9.

Figure 16 shows the cumulative error probability of localization for the different algorithms used in Scene 2. The experiments show that the proposed PSO-5DHLSTM-IPDR has the lowest cumulative localization error in the complex environment of Scene 2, which contains many metal structures and vehicles. This is due to the fact that the proposed algorithm can optimize the parameters of the hybrid neural network model using the particle swarm algorithm, which can enable the network model to still have a superior network performance in complex paths.

### 4.4. Mean Error and Root Mean Square Error

In this section, we verify the mean error and root mean square error of the proposed method in two scenes via different collection equipment.

Table 5 shows the mean error and root mean square error (RMSE) of the LSTM, MaLoc, PDR, DTW, and PSO-5DHLSTM-IPDR algorithms. The results show that the proposed algorithm can achieve a mean error of approximately 0.5 m and an RMSE of approximately 0.6 m. It is less than the localization error of all the other algorithms in the Table. This is because fused positioning mitigates geomagnetic positioning ambiguity and the cumulative error of pedestrian dead recognition.

Table 6 shows the mean errors and RMSEs of the different positioning methods for Scene 2 when different cell phones are used. The experimental results indicate that the presented PSO-5DHLSTM-IPDR algorithm can obtain smaller localization errors in a complex environment. It has good robustness and flexibility.

## 5. Conclusions

To overcome location ambiguity in geomagnetic localization and the accumulative error of dead reckoning, the PSO-5DHLSTM-IPDR localization method is proposed in this paper. First, in this localization method, we propose the PSO-5DHLSTM model, which optimizes the structural parameters of the 5DHLSTM neural network via the particle swarm algorithm. This approach can achieve more accurate geomagnetic localization. On the basis of the experimental results of PSO-5DHLSTM, the proposed method can achieve better positioning accuracy when different mobile phones are used. Moreover, we propose an improved PDR algorithm that extracts comprehensive heading features. An 8DBiLSTM network model is constructed for heading estimation in dead reckoning. Experiments demonstrate that the improved PDR can achieve better localization performances. Finally, the EKF is used to combine geomagnetic positioning and PDR positioning to achieve fusion positioning, which overcomes the disadvantages of single-positioning technologies. Extending experiments were carried out in two different scenarios: a return corridor with an area of 584 m^2^ and an underground car park with an area of 1393 m^2^. Two different mobile phones, Xiaomi 10 and Hi Nova 9, were used for the experiments. The return corridor was chosen because it simulates an environment with a regular structure but with some spatial variation, which is common in interiors. The scenario is very helpful in evaluating the performance of the localization algorithms in regular but varying indoor environments. Underground car parks are typically complex indoor environments with a number of disturbing factors. This scenario is chosen to test the localization accuracy and robustness of our method in the face of strong interference and complex spatial layouts, simulating the challenging environments that may be encountered in real applications. The experimental results of the proposed PSO-5DHLSTM-IPDR fusion positioning system show that good localization accuracy can still be maintained in both scenarios. It can be applied to different scenarios and devices with good robustness and flexibility.

## Figures and Tables

**Table 1:** Localization comparison between the 5DHLSTM method and the PSO-5DHLSTM method.

Method	Hidden Units	Learning Rate	Regular Rate	RMSE
5DHLSTM	100	0.004	0.04	1.5679
PSO-5DHLSTM	95	0.0097	0.0589	0.8624

**Table 2:** Mean error and RMSE of hierarchical LSTM, quaternion, and the proposed algorithm in Scene 1.

Scene 1	Method	Mean Error (°C)	RMSE (°C)
Xiaomi 10	Hierarchical LSTM	42.9333	83.5704
	Quaternion	18.6568	29.7198
	Proposed	10.463	39.6694
Hi Nova 9	Hierarchical LSTM	31.9899	86.0017
	Quaternion	55.9090	123.9004
	Proposed	10.1049	40.3392

**Table 3:** Mean error and RMSE of hierarchical LSTM, quaternion, and the proposed algorithm in Scene 2.

Scene 2	Method	Mean Error (°C)	RMSE (°C)
Xiaomi 10	Hierarchical LSTM	25.0119	70.2026
	Quaternion	73.8767	133.3659
	Proposed	18.9592	60.6685
Hi Nova 9	Hierarchical LSTM	37.5236	95.0320
	Quaternion	73.1892	143.9163
	Proposed	32.5796	85.4472

**Table 4:** Technical parameters of the phone.

Device	Hi Nova 9	Xiaomi 10
Operation system	Android	Android
CPU	Snapdragon 778G	Snapdragon 865
Batter capacity	4499 mAh	4780 mAh
RAM and ROM	8G + 128G	8G + 256G
Screen	7.0 inch	6.67 inch
Weight	175 g	208 g
Image resolution	2340 × 1080	2340 × 1080

**Table 5:** Mean errors and RMSEs of the LSTM, MaLoc, PDR, DTW, and PSO-5DHLSTM-IPDR algorithms in Scene 1 (m).

Scene 1	Method	Mean Error	RMSE
Xiaomi 10	LSTM	3.2381	4.4536
	MaLoc	2.7614	3.2397
	PDR	4.4760	4. 7955
	DTW	1.1059	1.2658
	PSO-5DHLSTM-IPDR	0.5049	0.5777
Hi Nova 9	LSTM	3.2434	4.4919
	MaLoc	4.1829	4.7916
	PDR	1.9446	2.2993
	DTW	2.0252	2.1274
	PSO-5DHLSTM-IPDR	0.5880	0.6867

**Table 6:** Mean errors and RMSEs of the LSTM, MaLoc, PDR, DTW, and PSO-5DHLSTM-IPDR algorithms in Scene 2 (m).

Scene 2	Method	Mean Error	RMSE
Xiaomi 10	LSTM	4.1927	5.7163
	MaLoc	1.2789	1.5590
	PDR	4.8835	5.4465
	DTW	1.3890	1.5340
	PSO-5DHLSTM-IPDR	0.5714	0.6381
Hi Nova 9	LSTM	4.1614	5.6468
	MaLoc	1.2715	1.5531
	PDR	1.5577	2.3503
	DTW	1.3596	1.5393
	PSO-5DHLSTM-IPDR	0.6411	0.7368

## Data Availability

The data that support the findings of this study are available on request from the corresponding author.

## References

[1] Uphaus P., Beringer B., Siemens K., Ehlers A., Rau H. (2021). Location-based services—The market: Success factors and emerging trends from an exploratory approach. J. Locat. Based Serv..

[2] Ogaja C.A. (2022). Introduction to GNSS Geodesy.

[3] Cao H., Wang Y., Bi J., Zhang Y., Yao G., Feng Y., Si M. (2024). LOS compensation and trusted NLOS recognition assisted WiFi RTT indoor positioning algorithm. Expert Syst. Appl..

[4] Shi T., Gong W. (2024). A Survey of Bluetooth Indoor Localization. arXiv.

[5] Al-Okby M.F.R., Junginger S., Roddelkopf T., Thurow K. (2024). UWB-Based Real-Time Indoor Positioning Systems: A Comprehensive Review. Appl. Sci..

[6] Huang L., Yu B., Li Y., Zhang H., Cheng J., Liang X., Liu S. (2024). High-precision Indoor Positioning Technology based on Multi-channel Array Pseudolite. IEEE Trans. Instrum. Meas..

[7] Huo G., Shang J., Zhang D., Wang Y. Geomagnetic Matching Combined Navigation Based on Improving Operation Efficiency of DTW Algorithm. Proceedings of the 2021 IEEE 15th International Conference on Electronic Measurement & Instruments (ICEMI).

[8] Subbu K.P., Gozick B., Dantu R. Indoor Localization through Dynamic Time Warping. Proceedings of the IEEE International Conference on Systems, Man and Cybernetics (SMC).

[9] Gong P., Wei D., Ji X., Li W., Yuan H. Research on Geomagnetic Matching Localization for Pedestrian. Proceedings of the China Satellite Navigation Conference (CSNC) 2018.

[10] Qiu K., Huang H., Li W., Luo D. Indoor geomagnetic positioning based on a joint algorithm of particle filter and dynamic time warp. Proceedings of the 2018 Ubiquitous Positioning, Indoor Navigation and Location-Based Services (UPINLBS).

[11] Xie H., Gu T., Tao X., Ye H., Lv J. MaLoc: A practical magnetic fingerprinting approach to indoor localization using smartphones. Proceedings of the 2014 ACM International Joint Conference on Pervasive and Ubiquitous Computing.

[12] Shu Y., Bo C., Shen G., Zhao C., Li L., Zhao F. (2015). Magicol: Indoor Localization Using Pervasive Magnetic Field and Opportunistic WiFi Sensing. IEEE J. Sel. Areas Commun..

[13] Khedr M.E., El-Sheimy N. (2020). SBAUPT: Azimuth SBUPT for frequent full attitude correction of smartphone-based PDR. IEEE Sens. J..

[14] Gusenbauer D., Isert C., Krösche J. Self-contained indoor positioning on off-the-shelf mobile devices. Proceedings of the 2010 International Conference on Indoor Positioning and Indoor Navigation.

[15] Wang J., Hu A., Liu C., Li X. (2015). A floor-map-aided WiFi/pseudo-odometry integration algorithm for an indoor positioning system. Sensors.

[16] Bae H.J., Choi L. Large-Scale Indoor Positioning using Geomagnetic Field with Deep Neural Networks. Proceedings of the ICC 2019—2019 IEEE International Conference on Communications (ICC).

[17] Bhattarai B., Yadav R.K., Gang H.-S., Pyun J.-Y. (2019). Geomagnetic Field Based Indoor Landmark Classification Using Deep Learning. IEEE Access.

[18] Lee N., Ahn S., Han D. (2018). AMID: Accurate Magnetic Indoor Localization Using Deep Learning. Sensors.

[19] Ashraf I., Kang M., Hur S., Park Y. (2020). MINLOC:Magnetic Field Patterns-Based Indoor Localization Using Convolutional Neural Networks. IEEE Access.

[20] Zhang M., Jia J., Chen J., Yang L., Guo L., Wang X. (2021). Real-time indoor localization using smartphone magnetic with LSTM networks. Neural Comput. Appl..

[21] Yang C.Y., Cheng Z.H., Jia X.X., Zhang L.T., Li L.Y., Zhao D.Q. (2023). A Novel Deep Learning Approach to 5G CSI/Geomagnetism/VIO Fused Indoor Localization. Sensors.

[22] Shu M., Chen G., Zhang Z., Xu L. (2023). Indoor Geomagnetic Positioning Using Direction-Aware Multiscale Recurrent Neural Networks. IEEE Sens. J..

[23] Ding X., Zhu M., Xiao B. Accurate Indoor Localization Using Magnetic Sequence Fingerprints with Deep Learning. Proceedings of the 21st International Conference on Algorithms and Architectures for Parallel Processing (ICA3PP).

[24] He T., Niu Q., He S., Liu N. Indoor Localization with Spatial and Temporal Representations of Signal Sequences. Proceedings of the IEEE Global Communications Conference (IEEE GLOBECOM).

[25] Wang Q., Jia J., Deng Y., Chen J., Wang X., Huang M., Aghvami A.H. (2024). DarLoc: Deep learning and data-feature augmentation based robust magnetic indoor localization. Expert Syst. Appl..

[26] Ni Y.-Z., Ni Z.-X. (2023). Geomagnetic indoor localisation-based dilated convolution neural networks. Nondestruct. Test. Eval..

[27] Liu F., Liu K., Guo X., Chen G., Zhou P., Yang J. Pedestrian Heading Estimation Based on F-LSTM Neural Network. Proceedings of the 2023 IEEE 16th International Conference on Electronic Measurement & Instruments (ICEMI).

[28] Wang Q., Luo H., Ye L., Men A., Zhao F., Huang Y., Ou C. (2019). Pedestrian Heading Estimation Based on Spatial Transformer Networks and Hierarchical LSTM. IEEE Access.

[29] Zhang M., Wen Y., Chen J., Yang X., Gao R., Zhao H. (2018). Pedestrian Dead-Reckoning Indoor Localization Based on OS-ELM. IEEE Access.

[30] Huang Y., Zeng Q., Lei Q., Chen Z., Sun K. Smartphone heading correction method based on LSTM Neural Network. Proceedings of the China Satellite Navigation Conference.

[31] Im C., Eom C., Lee H., Jang S., Lee C. Deep LSTM-Based Multimode Pedestrian Dead Reckoning System for Indoor Localization. Proceedings of the 2022 International Conference on Electronics, Information, and Communication (ICEIC).

[32] Ma Y., Dou Z., Jiang Q., Hou Z. (2016). Basmag: An optimized HMM-based localization system using backward sequences matching algorithm exploiting geomagnetic information. IEEE Sens. J..

[33] Kuang J., Niu X., Zhang P., Chen X. (2018). Indoor positioning based on pedestrian dead reckoning and magnetic field matching for smartphones. Sensors.

[34] Wu H., He S., Chan S.G. A graphical model approach for efficient geomagnetism-pedometer indoor localization. Proceedings of the 2017 IEEE 14th International Conference on Mobile Ad Hoc and Sensor Systems (MASS).

[35] Deng Z.-A., Hu Y., Yu J., Na Z. (2015). Extended Kalman filter for real time indoor localization by fusing WiFi and smartphone inertial sensors. Micromachines.

[36] Karamat T.B., Lins R.G., Givigi S.N., Noureldin A. (2018). Novel EKF-Based Vision/Inertial System Integration for Improved Navigation. IEEE Trans. Instrum. Meas..

[37] Sun M., Wang Y., Xu S., Qi H., Hu X. (2020). Indoor Positioning Tightly Coupled Wi-Fi FTM Ranging and PDR Based on the Extended Kalman Filter for Smartphones. IEEE Access.

[38] Schatzberg U., Banin L., Amizur Y. Enhanced WiFi ToF indoor positioning system with MEMS-based INS and pedometric information. Proceedings of the 2014 IEEE/ION Position, Location and Navigation Symposium—PLANS 2014.

[39] Wang Y., Li X. (2021). An improved robust EKF algorithm based on sigma points for UWB and foot-mounted IMU fusion positioning. J. Spat. Sci..

[40] Flandrin P., Rilling G., Goncalves P. (2004). Empirical mode decomposition as a filter bank. IEEE Signal Process. Lett..

[41] Huang N.E. A new method for nonlinear and nonstationary time series analysis: Empirical mode decomposition and Hilbert spectral analysis. Proceedings of the Conference on Wavelet Applications VII.

[42] Kuipers J.B. (1999). Quaternions and Rotation Sequences: A Primer with Applications to Orbits, Aerospace, and Virtual Reality.

[43] Zhong Z., Tang Z., Li X., Yuan T., Yang Y., Wei M., Zhang Y., Sheng R., Grant N., Ling C. XJTLUIndoorLoc: A New Fingerprinting Database for Indoor Localization and Trajectory Estimation Based on Wi-Fi RSS and Geomagnetic Field. Proceedings of the 6th International Symposium on Computing and Networking (CANDAR)—Across Practical Development and Theoretical Research.

[44] Koshi K., Matsuo S., Tadokoro Y. (2000). Pedestrian tracking system using digital portable telephones. Electron. Commun. Jpn. Part III-Fundam. Electron. Sci..

[45] Luo H., Zhao F., Jiang M., Ma H., Zhang Y. (2017). Constructing an Indoor Floor Plan Using Crowdsourcing Based on Magnetic Fingerprinting. Sensors.

